# The risk of cancer in patients with rheumatoid arthritis taking tumor necrosis factor antagonists: a nationwide cohort study

**DOI:** 10.1186/s13075-014-0449-5

**Published:** 2014-09-30

**Authors:** Chun-Ying Wu, Der-Yuan Chen, Jui-Lung Shen, Hsiu J Ho, Chih-Chiang Chen, Ken N Kuo, Han-Nan Liu, Yun-Ting Chang, Yi-Ju Chen

**Affiliations:** Faculty of Medicine, School of Medicine, National Yang-Ming University, Taipei, Taiwan; Division of Gastroenterology, Taichung Veterans General Hospital, Taichung, Taiwan; Department of Public Health and Graduate Institute of Clinical Medicine, China Medical University, Taichung, Taiwan; Department of Life Sciences, National Chung-Hsing University, Taichung, Taiwan; Department of Allergy, Immunology and Rheumatology, Taichung Veterans General Hospital, Taichung, Taiwan; Institute of Microbiology and Immunology, Chung-Shan Medical University, Taiwan, Taiwan; Department of Dermatology, Taichung Veterans General Hospital, Taichung, Taiwan; Department of Dermatology, Taipei Veterans General Hospital, Taipei, Taiwan; College of Medicine, Taipei Medical College, Taipei, Taiwan; Institute of Population Health Sciences, National Health Research Institutes, Miaoli, Taiwan

## Abstract

**Introduction:**

The association between cancer and use of biologic therapy among rheumatoid arthritis (RA) patients remains controversial. We aimed to compare the relative risk of cancer development between RA patients taking tumor necrosis factor α (TNFα) antagonists and those taking nonbiologic disease-modifying anti-rheumatic drugs (nbDMARDs).

**Methods:**

We conducted a nationwide cohort study between 1997 and 2011 using the Taiwan National Health Insurance Research Database. The risk of newly diagnosed cancer was compared between patients starting TNF-α antagonists (biologics cohort) and matched subjects taking nbDMARDs only (nbDMARDs cohort). Cumulative incidences and hazard ratios (HR) were calculated after adjusting for competing mortality. Standardized incidence ratio (SIR) was calculated for cancer risk. Multivariate analyses were performed using Cox proportional hazards model.

**Results:**

We compared 4426 new users of TNF-α antagonists and 17704 users of nbDMARDs with similar baseline covariate characteristics. The incidence rates of cancer among biologics and nbDMARDs cohorts were 5.35 (95% confidence interval (CI) 4.23 to 6.46) and 7.41 (95% CI 6.75 to 8.07) per 1000 person-years, respectively. On modified Cox proportional hazards analysis, the risk of cancer was significantly reduced in subjects in biologics cohort (adjusted HR 0.63, 95% CI 0.49 to 0.80, *P* < .001), after adjusting for age, gender, disease duration, major co-morbidities, and prior use of DMARDs and corticosteroids. However, there was an increased risk for hematologic cancers in biologics cohort, yet without statistical significance. The effect of biologics was consistent across all multivariate stratified analyses and the association between biologics use and cancer risk was independent of dosage of concomitant nbDMARDs.

**Conclusion:**

These findings suggested that RA patients taking TNF-α antagonist are associated with a lower risk of cancer, but not for hematologic cancers, than RA patients taking nbDMARDs alone.

**Electronic supplementary material:**

The online version of this article (doi:10.1186/s13075-014-0449-5) contains supplementary material, which is available to authorized users.

## Introduction

Rheumatoid arthritis (RA) affects both patient psychology and physiology, including cardiovascular, musculoskeletal and respiratory systems, and leads to major comorbidities and mortality. RA is associated with certain types of cancer [1-5]. Several studies have indicated an increased incidence of lymphoma [1,3,5-7] and decreased incidence of colorectal and gastric cancer in RA patients [6]. Cancer risk may be related to disease severity and treatment options. For example, the risk of lymphoma is substantially increased in those with high disease activity [8]. On the contrary, non-steroidal anti-inflammatory drugs (NSAIDs) have been reported to be associated with a reduced risk of colon cancer [9,10]. Traditional disease-modifying anti-rheumatic drugs (DMARDs) may also increase the risk of malignancy. For example, discontinuation of methotrexate has been followed by the disappearance of lymphoma in some patients [11].

The introduction of biologic therapies to the management of RA has raised concerns about the risk of cancer, particularly with respect to anti-TNF therapies, due to the role of TNF in tumor progression and surveillance [12]. An experimental animal tumor model has shown that anti-TNF-α antibodies hinder the innate anti-tumor immune responses and promote the growth of immunogenic rat colon tumors that are rejected by immunocompetent untreated rats. It has been suggested that biologics dampen the immune response against normally regressing cancers, thereby fostering malignant growth [13]. The evidence for the association between cancer occurrence and biologic use is conflicting. Systematic review and meta-analyses of clinical trial data have revealed an increased short-term risk of certain cancers in patients taking TNF inhibitors [14,15]. On the contrary, a recent meta-analysis of 63 randomized controlled trials demonstrated no association between the use of biologics and cancer risk in RA patients [16,17]. To date, only a few observational studies have been conducted on this issue, with controversial results on the long-term cancer risk among users of biologics with RA [18,19].

Here, we aimed to compare the cancer risk among RA patients starting TNFα blocker and those taking nonbiologic disease modifying antirheumatic drug (nbDMARD) in a large cohort of patients with RA, based on a Taiwanese nationwide database.

## Methods

### Study design

We conducted a nationwide cohort study by retrieving all patients with a diagnosis of RA from Taiwan’s National Health Insurance Research Database (NHIRD). The NHIRD has been used extensively in epidemiologic studies in Taiwan [3,20]. In brief, it consists of detailed health care data from more than 25 million enrollees, representing more than 99% of Taiwan’s entire population. In this database, the diagnostic codes are in the format of the International Classification of Diseases, Revision 9, Clinical Modification (ICD-9-CM). Patients were diagnosed by board-certified physicians in the corresponding specialties. The accuracy of diagnosis of major diseases in the NHIRD, such as stroke and acute coronary syndrome, has been validated. Personal information including body weight, height, family history, laboratory examination results, lifestyle and habits such as smoking and alcohol use was not available from the NHIRD.

This study has been approved by the ethical review board of the Taichung Veterans General Hospital, Taichung, Taiwan. As the datasets used in this study consist of de-identified secondary data released to the public for research purposes, no consent was needed for the review by the ethical review board.

### Biologic therapy in the RA cohort

Biologics were first introduced for RA management in Taiwan in March 2003. Biologics available for RA treatment in Taiwan include TNF-α inhibitors (adalimumab, etanercept and golimumab) and chimeric anti-CD20 monoclonal antibody (rituximab). Other classes of biologics, including humanized IL-6 receptor antibody (tocilizumab, or Actemra®) and selective T-cell co-stimulatory modulator (abatacept, or Orencia®), were not covered under the NHI program until mid 2012. Golimumab was not introduced to Taiwan until 1 January 2012. Therefore, none of the patients included in the present study were treated with golimumab, tocilizumab or abatacept.

Under the NHI program, only RA patients with continuous and active disease (fulfilling 1987 American College of Rheumatology (ACR) criteria for RA [21], with disease activity score 28-joint assessment (DAS 28) >5.1 points at least twice, recorded at least one month apart, and with radiologic and laboratory evidence) and who failed to respond to at least 6 months treatment with more than two nbDMARDs (mainly methotrexate, cyclosporine, hydroxychloroquine, D-penicillamine, gold salts, et cetera), can apply for reimbursement for biologics. Patients with prior history of pre-malignant or malignant diseases in the past 10 years or those with active infection are not eligible for biologics [22]. Information on medications was retrieved from the pharmacy prescription database. Reliability of the retrieved information was independently verified by two statisticians.

### Study cohorts

All patients with a primary diagnosis of RA (ICD-9-CM code 714.0) for the first time and who received nbDMARDs or biologics, including TNFα antagonists and rituximab, between 1997 and 2011 were eligible study subjects. The diagnostic accuracy of RA was confirmed by both specific ICD-9 codes and inclusion in the Registry for Catastrophic Illness Patient Database (RCIPD), a subpart of the NHIRD. As previously described, clinical and laboratory confirmation or typical image presentation of RA is required for patients to be registered in the RCIPD [3].

### Identification and definition of study groups

Patients who received TNFα antagonist, adalimumab or etanercept, or rituximab for RA, were eligible for inclusion in the biologics group. Patients taking nbDMARDs and who had never received prescription for biologics were eligible for inclusion in the nbDMARDs group. We created a matched sample by matching biologics and nbDMARDs subjects by date of birth, age at first use of DMARDs, gender, concomitant comorbidities, duration of disease, and starting date of study, as described below.

Follow up began on the date that a patient added or switched to a TNF-α antagonist (adalimumab or etanercept; golimumab was not yet available in 2011 in Taiwan) or rituximab. A matched calendar date as the index date was determined in matched nbDMARD subjects. Only when the index date is determined can we include those having no history of malignancies before or 6 months after the index date from the eligible nbDMARDs cohort. Finally, we selected matched patients in the nbDMARDs cohort fulfilling all of these criteria, including age, gender, comorbidities and those listed above. All participants were observed for the occurrence of outcomes, until death, or 31 December 2011. To study the patients with active disease requiring long-term disease control, we included only those who received nbDMARDs or biologics for at least 3 months after the start of follow up (the index date). Patients receiving follow up for less than 6 months were not included. Patients with history of malignant disease (ICD-9-CM codes: 140–208, ICD-O-3 codes: C00-C80) before or during the 6 months following the index date were not included.

### Main outcome measurements

Patients with cancer were defined as those having a new diagnosis of cancer (ICD-9-CM codes: 140–208, ICD-O-3 codes: C00-C80) at least 6 months after the index date. To reduce the mixed effect of prior use of nbDMARDs and new biologics, diseases of outcome diagnosed during the first 6 months of observation were not included.

### Identification of cancer cases

We identified the diagnoses of cancers based on the records of the RCIPD. To apply for a cancer catastrophic illness certificate, cytological or pathological reports or evidence including additional laboratory and imaging studies supporting the diagnosis of cancer, such as tumor marker surveys, radiograph, bone scan, computer tomography (CT) scan or magnetic resonance imaging (MRI) scan, must be provided. At least two other oncologists carefully examine the medical records and laboratory information including imaging studies. Only those patients who meet the criteria of diagnoses are issued certificates. We excluded those with *in-situ* malignancies, as *in-situ* malignant diseases do not qualify for a catastrophic illness certificate. The diagnostic codes of malignancies were defined as those from 140 to 208.91 in the ICD-9 revision clinical modification format (ICD-O-3 codes: C00-C80). We categorized the cancer cases into hematologic cancers and non-hematologic cancers. Hematologic cancers were subcategorized into leukemias (ICD9-CM codes 204 to 208; ICD-O3 codes: 9811 to 9818, 9820, 9823, 9826, 9827, 9831 to 9837, 9840, 9860 to 9861, 9863, 9865 to 9867, 9869, 9870 to 9876, 9891, 9895 to 9898, 9910, 9911, 9920, 9930, 9945, 9946, 9963, 9742, 9800, 9801, 9805 to 9809, 9931, 9940, 9948, 9964) and lymphomas (including non-Hodgkin’s lymphoma, multiple myeloma (ICD9-CM codes 200, 202 to 203; ICD-O-3 codes 9590, 9591, 9596, 9597, 9670, 9671, 9673, 9675, 9678 to 9680, 9684, 9687 to 9691, 9695, 9698, 9699, 9701, 9702, 9705, 9708, 9709, 9712, 9714, 9716 to 9719, 9724 to 9729, 9735, 9737, 9738, 9732 to 9733) and Hodgkin’s lymphoma (ICD9-CM code 201; ICD-O-3 codes 9650 to 9655, 9659, 9663 to 9665, 9667)), according to the methods of the Cancer Registry in Taiwan.

### Potential confounders

Certain demographic factors, such as age at first use of nbDMARDs, gender, and comorbidities such as hypertension, ischemic heart disease, including myocardial infarction, diabetes, cerebrovascular disease, and chronic liver disease, including liver cirrhosis, were considered potential confounders. These variables were determined over a one-year period before the start of follow up.

Other confounders included use of nbDMARDs, use of corticosteroids, and use of NSAIDs including aspirin, one year prior to the index date, as listed in Table 1. The use of statins and metformin have been reported to affect the development of certain cancers [23,24], and were also considered covariates.

**Table 1:** **Demographic characteristics of matched study cohorts**

**Characteristics**	**Biologics**	**nbDMARDs**	***P*** **-value**
	**(n =4,426)**	**(n =17,704)**	
Age, y, mean (SD)	53.88 (13.08)	53.89 (13.09)	0.97
Female gender, n (%)	3813 (86.20)	15252 (86.20)	1.00
Disease duration before index date, y, median (Q1, Q3)	9.21 (6.84, 11.23)	9.20 (6.81, 11.20)	0.70
Follow-up duration, y, median (Q1, Q3)	3.30 (2.00, 5.39)	3.25 (1.95, 5.29)	0.14
Outcome			
Cancer, n (%)	89 (2.00)	486 (2.70)	0.001
Death before outcome, n (%)	192 (4.30)	933 (5.30)	<0.001
Overall observation person-years	16650.63	65587.93	
Cancer, incidence rates (95% CI) per 1000 person-years	5.35 (4.23, 6.46)	7.41 (6.75, 8.07)	<0.005
Number of visits per year during follow ups, median (Q1, Q3)	13.33 (11.61, 14.81)	6.04 (3.15, 9.3)	<0.001
Co-morbidities, n (%)			
Hypertension	1418 (32.0)	5672 (32.0)	1.00
Chronic liver disease	813 (18.4)	3252 (18.4)	1.00
Ischemic heart disease	496 (11.2)	1984 (11.2)	1.00
Diabetes	388 (8.8)	1552 (8.8)	1.00
Cerebrovascular disease	96 (2.2)	384 (2.2)	1.00
Prior drug use, n (%)^1^			
Methotrexate	4125 (93.2)	8659 (48.9)	<0.001
Sulfasalazine	3276 (74.0)	8531 (48.2)	<0.001
Hydroxychloroquine	3515 (79.4)	10976 (62.0)	<0.001
Glucocorticosteroids	4003 (90.4)	12875 (72.7)	<0.001
Other systemic drugs use, n (%)^2^			
Statin	433 (9.8)	2069 (11.7)	<0.001
Metformin	264 (6.0)	1096 (6.2)	0.60
NSAID	4349 (98.3)	17041 (96.3)	<0.001
Beta-blockers	1115 (25.2)	4337 (24.5)	0.35
Average dosage of certain DMARDs during follow up, each user, mean (SD)^3^			
Methotrexate	9.1(4.32)	6.25 (4.31)	<0.001
Sulfasalazine	822.11(652.64)	720.69 (575.53)	<0.001
Hydroxychloroquine	194.71(137.37)	187.2 (123.95)	<0.001
Glucocorticosteroids	4.42 (3.6)	3.02 (3.21)	<0.001

^1^Drug users indicate patients using drugs within one year prior to the index date. ^2^Use of drugs at least once per month on average during follow up. ^3^The average dosage is depicted as mg/day for all these drugs, except methotrexate (mg/week). DMARDs, disease modifying anti-rheumatic drugs; N, number; NSAID, non-steroidal anti-inflammatory drugs including aspirin and Cox-2 inhibitors; Q, quartile.

No information on several potential confounders was available, such as smoking, alcohol use, family history, body mass index, rheumatoid arthritis activity, laboratory status, and educational level.

### Statistical analysis

The demographic data of the study population were first analyzed. Follow up for each subject was measured in numbers of years and began on the date of first prescription of biologics in the biologics group (or the matching calendar date in the nbDMARDs group) and ended on the date of censorship, that is, the date of diagnosis of outcome, death, transfer out or the end of the follow-up period.

As death may result from underlying illness, which may also affect the outcome, it leads to informative censoring in the estimation of the incidence of outcome diseases. Therefore, death occurring prior to outcome was considered a competing risk event. The cumulative incidence of newly diagnosed cancers after adjustment for competing mortality was calculated using a two-step process and tested for equality among the study cohorts. Calculation and comparison of cumulative incidence in the presence of competing risk data ratios was conducted using a modified Kaplan-Meier method and Gray’s method [25]. We tested the differences in the full time-to-event distributions between the study groups using the log-rank test. The assumption of proportional hazards was confirmed by plotting the graph of the survival function versus the survival time and the graph of the log (−log(survival)) versus the log of survival time.

We applied a modified Cox proportional hazards model in the presence of a competing risk event to examine the independent association between newly diagnosed cancers and use of biologics [25,26]. Assessment of goodness-of-fit of the models with the step-down method was carried out to analyze the independent risk factors. The influence of biologics on newly diagnosed cancer was further explored by stratification according to age, gender, disease duration, time from start of follow up, and prior use of DMARDs or corticosteroids.

Sensitivity analyses included (1) focusing on individuals who used adalimumab only; ever used adalimumab; or last use of adalimumab before cancer occurrence; and (2) focusing on individuals who used etanercept only; ever used etanercept; or last use of etanercept before cancer occurrence. These sensitivity analyses were conducted with the purpose of examining whether the main findings were robust to different assumptions.

All data management was performed using SAS 9.2 software (SAS Institute Inc., Cary, NC, USA). Calculations of cumulative incidences and Cox models were carried out using the *cmprsk* package of R [27]. Calculated results were expressed as the estimated number together with the 95% CI.

## Results

### Demographic characteristics of study cohorts

**Figure 1:**
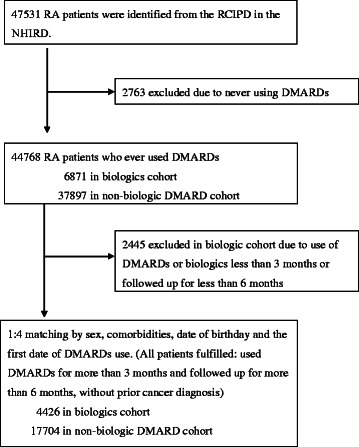
**Flow chart of study subject selection.** RA, rheumatoid arthritis; RCIPD, Registry for Catastrophic Illness Patient Database; NHIRD, Taiwan National Health Insurance Research Database; DMARD, disease-modifying anti-rheumatic drug.

We identified 47,531 potentially eligible RA patients from the RCIPD. A total of 2,763 patients who never received DMARDs were excluded. Among the remaining 44,768 subjects, 6,871 patients with a history of biologics use including TNFα antagonists and rituximab were eligible for inclusion in the biologics group and the remaining 37,897 patients who had never used biologics were eligible to be included in the nbDMARDs group. We excluded 2,445 patients in the eligible biologics group who received biologics or traditional DMARDs for less than 3 months; or were followed up for less than 6 months, after starting biologics treatments. Next, we matched four subjects in the eligible nbDMARDs cohort with each subject in the biologics cohort, based on the matching criteria listed in Methods. Finally, the biologics group and the nbDMARDs group consisted of 4,426 and 17,704 patients, respectively, as shown in Figure 1.

The biologics group and nbDMARDs group were similar in demographic characteristics and associated comorbidities (Table 1). In the biologics group, 3,270 patients (73.9%) received etanercept, 1,577 patients (35.6%) received adalimumab and 578 patients (13.1%) received rituximab. There were 2,529 patients who received etanercept only, 996 patients who received adalimumab only, and 10 patients who received rituximab only. It is not uncommon for biologics to be switched. For example, 323 patients switched from adalimumab to etanercept; 310 patients switched from etanercept to rituximab; 150 patients switched from adalimumab to rituximab; and 108 patients switched treatment among all three biologics.

Disease duration, mean observation time, and number of hospital visits are presented in Table 1. Subjects in the biologics group took more DMARDs and corticosteroids than those in the nbDMARDs group before the index date (Table 1). In addition, more than 92% of patients in the biologics group received biologics in combination with nbDMARDs or corticosteroids after the index date. The average daily dosages of combined nonbiologic DMARDS in the biologics group were higher than in the nbDMARDs group (Table 1, Additional file 1: Table S1).

### Incidence rates of newly diagnosed cancers

**Figure 2:**
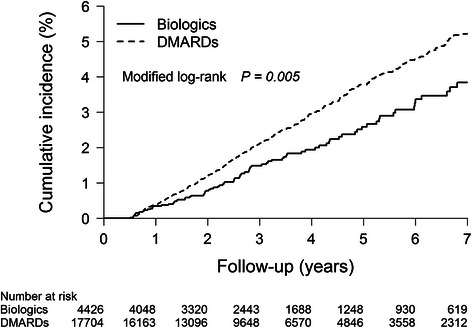
**Cumulative incidences of new cancer occurrence following initiation of biologics after adjustment for competing mortality.** Calculation and comparison of cumulative incidences in the presence of competing risk data ratios were conducted using a modified Kaplan-Meier method and Gray’s method. New cancer occurrence during the first six months was excluded. DMARDs, disease-modifying anti-rheumatic drugs.

A total of 89 patients in the biologics group and 486 patients in the nbDMARDs group presented with newly diagnosed cancer during the observation period. The 7-year cumulative incidence of newly diagnosed cancer after adjusting for competing mortality was significantly lower in the biologics group (3.84%, 95% CI 2.91, 4.77) than in the nbDMARDs group (5.22%, 95% CI 4.69, 5.75) (*P* =0.005), as shown in Figure 2.

The incidence rates of newly diagnosed cancer were estimated to be 5.35 per 1000 patient-years (95% CI 4.23, 6.46) in the biologics group and 7.41 per 1000 patient-years (95% CI 6.75, 8.07) in the nbDMARDs group, with statistically significant difference (Table 2). No cancer cases were observed among those taking rituximab alone. The cancer risk was lower in those taking adalimumab alone, when compared with those taking etanercept alone, but without statistical significance.

**Table 2:** **Multivariate analyses of risk of malignant diseases among biologic users in comparison with matched controls**

		**All**	**TNF-α** **inhibitor** ^**1**^ **users**	**Adalimumab** ^**2**^	**Etanercept** ^**3**^	**Rituximab**
Biologics	Event, number	89	89	8	71	0
	Total person-years	16650.63	16624.36	2203.45	10363.75	26.27
	IR per 1000 (95% CI)	5.35 (4.23, 6.46)	5.35 (4.24, 6.47)	3.63 (1.11, 6.15)	6.85 (5.26, 8.44)	-
nbDMARDs^4^	Event, number	486	485	51	309	1
	Total person-years	65587.93	65485.91	8763.78	41209.78	102.02
	IR per 1000 (95% CI)	7.41 (6.75, 8.07)	7.41 (6.75, 8.07)	5.82 (4.22, 7.42)	7.50 (6.66, 8.33)	9.80 (0.00, 29.01)
	HR (95% CI)	0.63 (0.49, 0.80)	0.63 (0.50, 0.81)	0.61 (0.28, 1.33)	0.83 (0.62, 1.10)	-
	IRR (95% CI)	0.72 (0.58, 0.90)	0.72 (0.58, 0.91)	0.62 (0.30, 1.31)	0.91 (0.71, 1.18)	-
	*P*-value	0.0046	0.0049	0.2147	0.4926	-

^1^Includes all patients who have used TNF-α inhibitors, with or without disease-modifying anti-rheumatic drugs (DMARDs) or corticosteroids, in comparison with matched controls. ^2^Use of adalimumab alone, with or without DMARDs or corticosteroids, in comparison with matched controls. ^3^Use of etanercept alone, with or without DMARDs or corticosteroids, in comparison with matched controls. ^4^Indicates the event number and total observed person-years of matched subjects from THE nonbiologic DMARDS (nbDMARDs) cohort for each of those using different types of TNF-α inhibitors and rituximab. HR, hazard ratio; IR, incidence rate; IRR, incidence rate ratio.

### Multivariate analysis after adjustment for competing mortality

When compared with the use of nbDMARDs, starting of TNF-α antagonists was associated with a significantly reduced risk of cancer (adjusted HR 0.63, 95% CI 0.49, 0.80, *P* <0.0001), after adjusting for age, gender, disease duration, number of hospital visits, prior use of DMARDs, prior use of systemic corticosteroids, and presence of diabetes, hypertension, ischemic heart disease, cerebrovascular disease, or chronic liver disease (Table 3).

**Table 3:** **Death-adjusted multivariate analyses of cancer risk in both study cohorts**

**Covariate**	**Adjusted hazard ratio** ^**4**^	**95%** **CI**	***P*** **-value**
Biologics use	0.63	0.49, 0.80	<0.001
Male gender	1.10	0.88, 1.38	0.39
Age at start, per year	1.04	1.03, 1.05	0.000
Disease duration^1^, per year	0.96	0.94, 0.99	0.02
Number of hospital visits, per year during observation	1.01	1.01, 1.02	0.000
Methotrexate^2^	1.04	0.86, 1.26	0.68
Salfulsalazine^2^	1.01	0.84, 1.20	0.93
Hydroxychloroquine^2^	1.13	0.94, 1.36	0.19
Corticosteroids^2^	1.03	0.84, 1.28	0.75
Diabetes mellitus	1.26	0.92, 1.74	0.15
Ischemic heart disease	0.70	0.54, 0.92	0.01
Cerebrovascular disease	0.75	0.45, 1.26	0.28
Hypertension	1.21	0.98, 1.50	0.07
Chronic liver disease	1.16	0.94, 1.42	0.17
Statins^3^	0.64	0.48, 0.86	0.003
Metformin^3^	0.67	0.45, 1.00	0.05
NSAIDs^3^	0.94	0.54, 1.64	0.83
Beta-blocker^3^	0.77	0.63, 0.93	0.008

^1^Disease duration represented by duration from the first disease-modifying anti-rheumatic drugs (DMARDs) prescription for rheumatoid arthritis to the index date. ^2^Use of drugs within one year prior to the index date. ^3^Use of drugs at least once per month on average during follow-up. ^4^Modified hazard ratio adjusted by multiple covariates including age, gender, disease duration, prior history of hypertension, diabetes, ischemic heart disease, cerebrovascular disease, chronic liver diseases, use of DMARDs within one year prior to the index date, corticosteroids, aspirin, non steroidal anti-inflammatory drugs (NSAIDs), statins and metformin.

Use of statins, use of metformin, use of beta-blockers, long disease-duration and presence of ischemic heart disease were also associated with a lower risk of cancer. On the contrary, increasing age and higher average number of hospital visits were significantly associated with cancer occurrence (Table 3).

### Multivariate stratified analyses after adjustment for competing mortality

**Figure 3:**
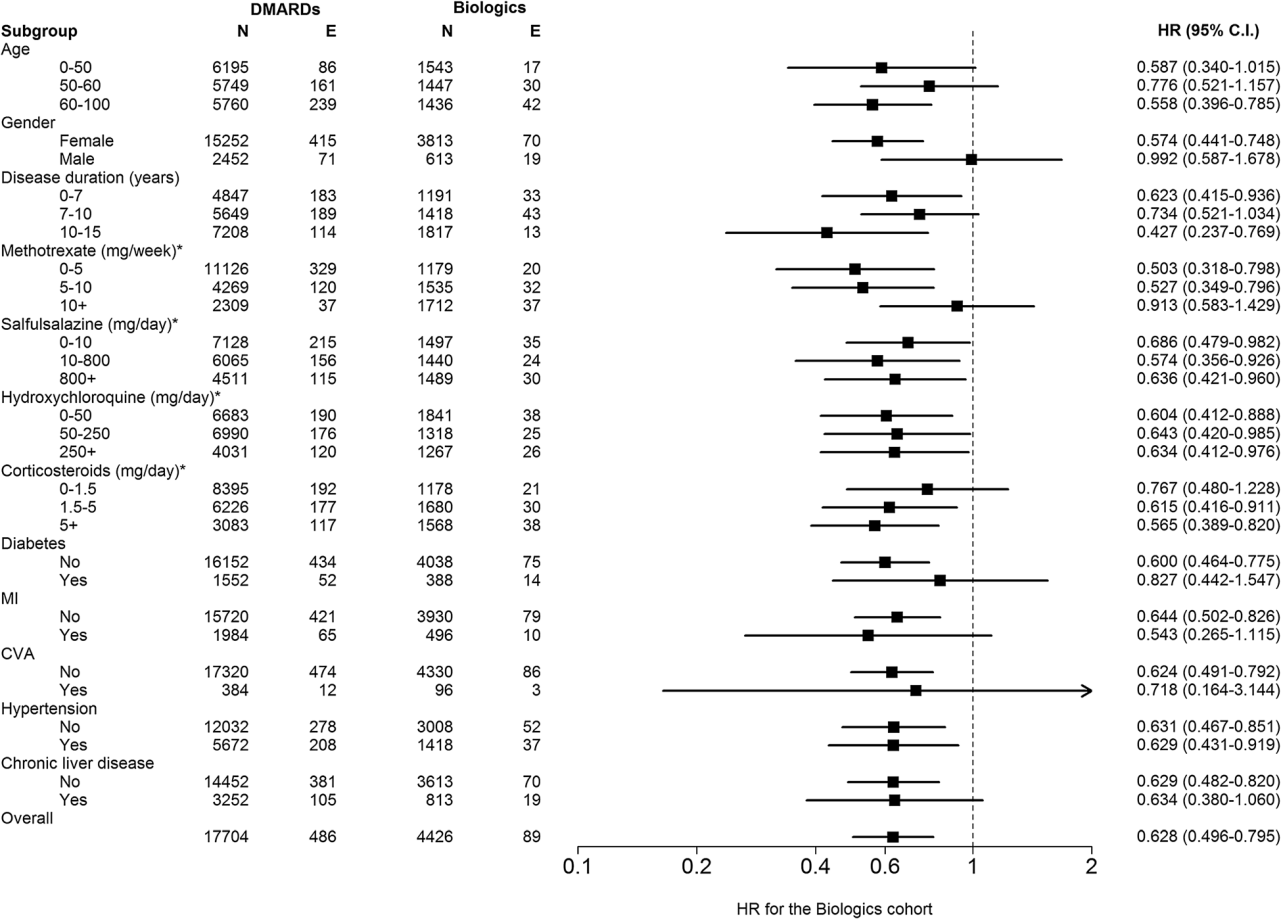
**Multivariate stratified analyses for cancer occurrence between biologics cohort and biologics-naive cohort.** All analyses were stratified according to age, gender, disease duration, average dosage of corticosteroids or disease-modifying anti-rheumatic drugs (DMARDs) after the index date, and coexisting comorbidities, based on a modified Cox proportional hazards model in the presence of a competing risk event. Some hazard ratios (HR) were not statistically significant due to a small number of cases. *Indicates average drug use after the index date of observation. Event, new cancer occurrence; MI, ischemic heart disease, including myocardial infarction; N, number; CVA, cerebrovascular attack.

We conducted multivariate stratified analyses adjusted for competing mortality to determine the effects of biologics on cancer development among different subsets of RA patients. A negative association between biologics use and cancer occurrence was observed in almost all subsets of study subjects, especially among those of older age, of female gender, with long-disease duration, and free of comorbidities (Figure 3).

To examine the potential mixed effect of DMARDs or corticosteroids on cancer risk in biologics users, we further conducted analyses by stratifying the dose of concomitant DMARDs. The association between biologics use and cancer risk was independent of daily dosage of concomitant nbDMARDs.

### Specific cancer risks and sensitivity analyses by standardized incidence ratio (SIR)

Sensitivity analyses focusing on patients receiving different treatment patterns of TNF-α inhibitors is presented in Additional file 2: Table S2. We performed SIR estimation to compare the cancer risks of both study groups with the general population. The cancer risk was significantly elevated among subjects in the nbDMARDs cohort when compared with the general population, both for non-hematologic cancers (SIR 1.31, 95% CI 1.19, 1.44) and hematologic cancers (SIR 2.28, 95% CI 1.55, 3.24) (Table 4). On the contrary, the overall cancer risk for subjects in the biologics cohort was comparable to that of the general population (SIR 0.97, 95% CI 0.78, 1.19). The overall cancer risk in those taking adalimumab was slightly lower than in those taking etanercept, either in the patterns of treatment alone, ever-treated or last-treated with the biologics of interest, yet without statistical significance (Table 4, Additional file 2: Table S2).

**Table 4:** **Standardized incidence ratio (SIR) of specific cancer types among subjects in the biologics cohort and nonbiologic DMARDs cohort**

**Cancer origin**	**Biologics**		**SIR (95%** **CI)**	**nbDMARDs**		**SIR (95%** **CI)**
	**N**	**Expected**		**N**	**Expected**	
**Non-hematologic cancers**	**73**	**88.46**	**0.83 (0.65, 1.04)**	**455**	**347.47**	**1.31 (1.19, 1.44)**
Oral cavity	2	4.55	0.44 (0.05, 1.59)	20	17.92	1.12 (0.68, 1.72)
Digestive system^1^	20	32.03	0.62 (0.38, 0.97)	135	125.75	1.07 (0.90, 1.27)
Lungs^2^	11	10.39	1.06 (0.53, 1.89)	57	40.77	1.40 (1.06, 1.81)
Breast, cervix^3^	22	19.96	1.10 (0.69, 1.67)	115	78.45	1.47 (1.21, 1.76)
Bladder/kidney^4^	14	15.75	0.89 (0.49, 1.49)	96	61.81	1.55 (1.26, 1.90)
Melanoma	0	0.21	-	0	0.83	-
NMSC	5	2.43	2.05 (0.66, 4.79)	15	9.58	1.57 (0.88, 2.58)
Unspecified origin	4	5.79	0.69 (0.19, 1.77)	32	22.76	1.41 (0.96, 1.99)
**Hematologic cancers** ^**5**^	**16**	**3.45**	**4.64 (2.65, 7.53)**	**31**	**13.57**	**2.28 (1.55, 3.24)**
Lymphoma	13	2.12	6.13 (3.26, 10.49)	21	8.34	2.52 (1.56, 3.85)
NHL	12	2.05	5.86 (3.02, 10.24)	20	8.06	2.48 (1.52, 3.83)
HL	1	0.07	13.97 (0.18, 77.7)	1	0.28	3.56 (0.05, 19.79)
Leukemia	1	1.33	0.75 (0.01, 4.18)	9	5.23	1.72 (0.79, 3.27)

^1^Cancers of digestive system include cancers of the stomach, colon, liver, pancreas. ^2^Including cancers of the lungs, bronchus and pleura. ^3^Including cancers of the uterus and other female reproductive organs. ^4^Including cancers of the prostate, testis and other male reproductive organs. ^5^Myeloma not included. NbDARDS, nonbiologic disease-modifying anti-rheumatic drugs; HL, Hodgkin’s lymphoma; NHL, non-Hodgkin’s lymphoma; NMSC, non-melanoma skin cancers; N, number.

However, the risk of hematologic cancer was significantly elevated in the biologics cohort when compared with the general population (SIR 4.64, 95% CI 2.65, 7.53) (Table 4). The risk of non-Hodgkin’s lymphoma was comparable between those taking adalimuamb and those taking etanercept, either in the patterns of treated alone, ever-treated or last-treated with the biologics of interest, without statistical significance (Additional file 2: Table S2).

## Discussion

Our study provided nationwide-based evidence that starting TNF-α antagonists does not increase, but rather decreases, the risk of cancer for RA patients, in comparison with those taking nbDMARDs alone, with a reduction in 7-year cumulative incidence from 5.22% to 3.84%. The effect of TNF-α antagonists was statistically significant among almost all subgroups of patients. The association between TNF-α antagonists and cancer risk was independent of daily dosage of concomitant nbDMARDs.

Controversy remains with respect to whether biologics carry a cancer risk for RA patients. A meta-analysis that included nine RCTs and 5,005 subjects conducted by Bongartz *et al*. [15] demonstrated a dose-dependent increased risk of malignancy in RA patients treated with TNF inhibitors, mainly infliximab and adalimumab. On the contrary, the most recent systemic review and meta-analysis from 63 RCTs that included 29,423 patients revealed no statistically significant increased risk of any type of cancer with use of TNF antagonists plus nbDMARDs, in comparison with use of nbDMARDs alone [16]. The results from the British Society for Rheumatology Biologics Registry for RA patients demonstrated no difference in risk of solid cancer among patients receiving TNF-α antagonists and patients receiving nbDMARDs over a 5-year observation period. That study also demonstrated no difference in the relative risk of cancer for any of the individual TNF-α antagonists [28]. Two small clinical trials in Taiwan reported a negative association with short-term (12-week) risk for cancer in RA patients using etanercept and adalimumab, compared to RA patients taking methotrexate [29,30]. Several other clinical trials have reported controversial results for long-term cancer risk in subjects taking TNF-α antagonists, with an observation time of up to 104 weeks [31-35]. However, half of the trials only included subjects with active early RA (less than 2 years).

The association between lymphoma occurrence and use of biologics in RA has been of major concern [7,17,36,37]. In the present study, we demonstrated a significantly elevated risk of lymphoma in both the biologics cohort and nbDMARDs cohort when compared with the general population. Higher risk of lymphoma was observed in the biologics cohort when compared with the nbDMARDs cohort, with statistical significance (Table 4, Additional file 2: Table S2). Data from the MedWatch postmarketing adverse event surveillance system of the Food and Drug Administration have revealed an association between lymphoma and treatment with etanercept or infliximab [38]. Recent meta-analyses of 71 global clinical trials investigating the safety profiles of adalimumab across several entities of arthritis have indicated a slightly increased risk of lymphoma among adalimumab users with RA when compared with the general population [39]. However, a study comparing published case reports and reports from the French pharmacovigilance system has argued the link between lymphoma and TNF-α antagonists [40]. A prospective cohort study with a large sample size and a long duration of follow up is required to examine the causality between biologics and cancer development.

The association between malignant disease and chronic inflammation is well-documented [41]. We assumed that patients taking biologics had more severe disease and were more likely to develop malignant diseases. On the contrary, the chance of developing cancer was reduced in patients taking biologics, in comparison with matched patients taking nbDMARDs only. This implies that the use of biologics attenuates the disease activity and then reduces the risk of cancer development to a level that is even lower than in subjects taking nbMDARDs only.

The mixed effect of concomitant DMARDs may also affect the risk of cancer. Physicians in Taiwan usually reserve low-dose DMARDs in combination with biologics for reducing the chance of auto-antibody production and improving clinical outcomes. Our subjects in the biologics cohort received a higher daily dosage of DMARDs than those in the matched nbDMARDs cohort (Table 1). These results suggested that patients in the biologics cohort have more severe or refractory disease than those in the nbDMARDs cohort. Further multivariate stratified analyses suggested that the association between biologics use and cancer risk is independent of dosage of concomitant non-biologic DMARDs. This further emphasizes the additional beneficial role of biologics in patients with severe RA.

A low prevalence of RA has been reported in Asian countries [42-44]. Based on the estimate from the Taiwan Rheumatology Association and nationwide epidemiologic studies, the prevalence of RA is between 0.2 and 0.4% of the Taiwanese general population [42]. Likewise, the prevalence of RA is less than 0.3% in China [43] and decreased from 0.54% in 1969 to 0.17% in 1996 in Japan [44]. These prevalence estimates are all much lower than the estimated 1% prevalence in Western countries. The estimate of prevalence in the present study (approximately 0.2%) is consistent with that of prior studies in Taiwan. However, as we included only patients fulfilling all diagnostic criteria for RA, some patients with very early RA, not registered in the RCIPD, may not have been included.

The incidence rates of cancer in RA have been reported to be 4.58 in Korea and 6.7 in Taiwan per 1000 patient-years, which are lower than in Western populations [45,46]. The incidence rates of all-site malignancies in TNF-α antagonist users in Western populations have been reported to range from 4.7 to 13 per 1000 patient-years [18,19,47]. More recently, Mercer *et al*. reported the incidence rate of cancer in TNF-α antagonist users to be 8.1 per 1000 patient-years, compared with 11.7 per 1000 patient-years in nbDMARDs users [28]. The results from the present study are consistent with those of prior studies. More epidemiologic studies are required to determine the cancer prevalence in more Asian populations.

On multivariate analysis, more hospital visits were positively associated with cancer occurrence. Patients with more hospital visits were supposed to have more chance of cancer surveillance. Although there were more hospital visits among subjects in the biologics cohort than the matched nbDMARDs cohort, as presented in Table 1, our results further reinforced the link between biologics use and a lower risk of cancer.

Patients with ischemic heart disease (IHD) were found to have a negative association with cancer occurrence on multivariate analyses. Patients with IHD tended to have fatal cardiovascular outcomes and might die too early to develop a new cancer. After matching comorbidities in both study cohorts, this bias may happen randomly in both cohorts. In addition, the medications commonly used for IHD may play a role in preventing certain cancer occurrence or recurrence [48-50]. The negative association is observed independently for use of statins, and NSAIDs, including aspirin or beta-blockers, on multivariate analyses. This link is worth further investigation. Different study designs would be needed to determine the role of other anti-hypertensive drugs on cancer risk.

The strengths of the current study include the utilization of the nationwide NHIRD, which contains detailed pharmacy claims for each study subject and is widely accepted for epidemiological studies [3,20,46]. To eliminate immortal time bias, we applied incident user design to this study, with the exposure time calculated from the start of new biologics in the biologics group (and corresponding date in the nbDMARDs group). In addition, we matched both biologics and comparator groups for age, gender, propensity score, date of first use of DMARDs, and date of first use of biologics. An active comparator cohort, as was used in this study, serves to restrict analyses to patients with active long-term disease of similar severity and with similar comorbidity index.

There are several limitations to the present study. It is difficult to infer causation between a drug of interest and risk of cancer occurrence based on an observational study, without a random assignment of treatments. Confounding by indication may exist and account for differences in outcomes. In addition, the patients in the study cohorts may differ in many measured and unmeasured ways. We did not have personal information such as lifestyle, family history of malignant diseases, body mass index, laboratory or serologic information, and records of disease severity score, all of which may contribute to cancer risk. To avoid these biases, we selected only patients with matched age, gender, disease duration, and concomitant comorbidities. Multivariable analysis was performed to adjust for potential confounders. Furthermore, we conducted multivariable stratified analysis to examine the risk of cancer for the study cohorts in different strata. Although unmeasured confounders may still exist, we believe the methodology used in the present study is solid and robust. To meet the eligibility for biologics use, we selected only subjects free of cancer before taking biologics or the start of follow up, which may have led to an underestimation of cancer incidence in both groups.

Coding error is possible in a database. To minimize this bias, we enrolled only patients from the catastrophic illness database who met the criteria for RA and malignant diseases. Furthermore, most patients undergo regular physical and laboratory examination during observation. A surveillance bias may contribute to an increased frequency of cancer. Our analyses were adjusted for number of hospital visits to prevent this bias. Some patients may have used self-paid biologics and thus may have been inappropriately classified into the nbDMARDs cohort. This potential misclassification may have led to an underestimation of the association.

Nonetheless, our study provides important evidence of the safety, in terms of cancer risk, of anti-TNF-α antagonists in RA patients who survive long-term disease and who undergo long-term DMARD treatment. In addition, the potential beneficial effect of biologics was most significant among those of female gender, those with long disease-duration and those free of major comorbidities. The association between biologics and cancer risk was independent of daily dosage of concomitant nbDMARDs.

The results of our study should be interpreted cautiously because they refer only to two out of five available TNF-α antagonists. The association between cancer risk and other TNF-α antagonists needs further analysis. Moreover, these results refer only to RA patients without prior malignant diseases. The investigation of the effect of biologics on patients with existing malignancy requires a different study design.

## Conclusion

The current study suggested that the addition of anti-TNF-α therapy is safe, in terms of cancer risk, for RA patients undergoing long-term DMARD treatment, based on a Taiwanese nationwide population.

## Additional files

Additional file 1: Table S1. Comparison of non-biologics disease-modifying anti-rheumatic drug use prior to the index date between the two study groups. Additional file 2: Table S2. Sensitivity analyses for the cancer risk among different biologics treatment settings.

## Abbreviations

ACR: American College of Rheumatology
DAS28: Disease activity score 28-joint assessment
DMARD: disease-modifying anti-rheumatic drug
HR: hazard ratio
ICD-9-CM: International Classification of Diseases, Revision 9, Clinical Modification
nbDMARD: nonbiologic disease-modifying anti-rheumatic drug
NHIRD: Taiwan national Health Insurance Research Database
NSAID: non-steroidal anti-inflammatory drug
RA: rheumatoid arthritis
RCIPD: Registry for Catastrophic Illness Patient Database
TNFα: tumor necrosis factor α
