# Erratum to: The risk of cancer in patients with rheumatoid arthritis taking tumor necrosis factor antagonists: a nationwide cohort study

**DOI:** 10.1186/s13075-016-1016-z

**Published:** 2016-05-17

**Authors:** Chun-Ying Wu, Der-Yuan Chen, Jui-Lung Shen, Hsiu J. Ho, Chih-Chiang Chen, Ken N. Kuo, Han-Nan Liu, Yun-Ting Chang, Yi-Ju Chen

**Affiliations:** Faculty of Medicine, School of Medicine, National Yang-Ming University, Taipei, Taiwan; Division of Gastroenterology, Taichung Veterans General Hospital, Taichung, Taiwan; Department of Public Health and Graduate Institute of Clinical Medicine, China Medical University, Taichung, Taiwan; Department of Life Sciences, National Chung-Hsing University, Taichung, Taiwan; Department of Allergy, Immunology and Rheumatology, Taichung Veterans General Hospital, Taichung, Taiwan; Institute of Microbiology and Immunology, Chung-Shan Medical University, Taiwan, Taiwan; Department of Dermatology, Taichung Veterans General Hospital, Taichung, Taiwan; Department of Dermatology, Taipei Veterans General Hospital, Taipei, Taiwan; College of Medicine, Taipei Medical College, Taipei, Taiwan; Institute of Population Health Sciences, National Health Research Institutes, Miaoli, Taiwan

Unfortunately, after publication of this article [1], it was noticed that there was an error in the funding information located in the Acknowledgements section. The support grants for the study should instead be listed as TCVGH-1026802C and TCVGH-1026801B, seen in the correct Acknowledgements below.
