# Supplementary material for: The risk of cancer in patients with rheumatoid arthritis taking tumor necrosis factor antagonists: a nationwide cohort study
Source: Arthritis Res Ther. 2014 Sep 30;16:449. doi: 10.1186/s13075-014-0449-5 (PMC4201718; doi:10.1186/s13075-014-0449-5)
Supplement: Additional file 1: Table S1. — Comparison of non-biologics disease-modifying anti-rheumatic drug use prior to the index date between the two study groups. [file 13075_2014_449_MOESM1_ESM.doc]

Supplementary Table 1. Comparison of non-biologics disease modifying anti-rheumatic drugs use prior to the index date between the two study groups

| Average dosage of certain DMARDs during follow up, each user*, mean (SD) | Biologics cohort | nbDMARDs cohort | P value |
| --- | --- | --- | --- |
| Methotrexate | 9.1(4.32) | 6.25 (4.31) | <.001 |
| Sulfasalazine | 822.11(652.64) | 720.69 (575.53) | <.001 |
| Hydroxychloroquine | 194.71(137.37) | 187.2 (123.95) | <.001 |
| Glucocorticosteroids | 4.42 (3.6) | 3.02 (3.21) | <.001 |
| Average dosage of certain DMARDs prior to index date, each user*, mean (SD) |  |  |  |
| Methotrexate | 10.1 (3.76) | 6.71(4.18) | <.001 |
| Sulfasalazine | 1242.78 (625.89) | 834.4 (596.55) | <.001 |
| Hydroxychloroquine | 276.74 (108.92) | 208.89 (123.5) | <.001 |
| Glucocorticosteroids | 6.15 (6.66) | 3.68 (3.73) | <.001 |

Abbreviations: nbDMARDs, non-biologic disease modifying anti-rheumatic drugs; SD, standard deviation

* Indicates the average dosages only for users, not including all patients.
