# Supplementary material for: The risk of cancer in patients with rheumatoid arthritis taking tumor necrosis factor antagonists: a nationwide cohort study
Source: Arthritis Res Ther. 2014 Sep 30;16:449. doi: 10.1186/s13075-014-0449-5 (PMC4201718; doi:10.1186/s13075-014-0449-5)
Supplement: Additional file 2: Table S2. — Sensitivity analyses for the cancer risk among different biologics treatment settings. [file 13075_2014_449_MOESM2_ESM.doc]

Supplementary Table 2. Sensitivity analyses for the cancer risk among different biologics treatment settings.

| **Cancer Origins** | **Observed** | **Expected** | **SIR (95% CI)** |
| --- | --- | --- | --- |
| **Overall cancers** |  |  |  |
| Biologics, all | 89 | 91.91 | 0.97 (0.78-1.19) |
| Adalimumab^¶^ | 16 | 27.04 | 0.59 (0.34-0.96) |
| Adalimumab§ | 11 | 20.31 | 0.54 (0.27-0.97) |
| Adalimumab† | 8 | 12.64 | 0.63 (0.27-1.25) |
| Etanercept^¶^ | 79 | 76.75 | 1.03 (0.82-1.28) |
| Etanercept§ | 73 | 58.24 | 1.25 (0.98-1.58) |
| Etanercept† | 71 | 56.18 | 1.26 (0.99-1.59) |
| **Non-Hodgkin’s Lymphoma** |  |  |  |
| Biologics, all | 12 | 2.05 | 5.86 (3.02-10.24) |
| Adalimumab^¶^ | 3 | 0.60 | 4.96 (1.00-14.49) |
| Adalimumab§ | 2 | 0.46 | 4.39 (0.49-15.85) |
| Adalimumab† | 2 | 0.28 | 7.05 (0.69-25.46) |
| Etanercept^¶^ | 9 | 1.71 | 5.27 (2.40-10.00) |
| Etanercept§ | 8 | 1.29 | 6.18 (2.66-12.18) |
| Etanercept† | 8 | 1.32 | 6.40 (2.76-12.62) |

Abbreviations: CI, confidence interval; Exp, expected number; nbDMARDs, non-biological disease modifying anti-inflammatory drugs ;SIR, standardized incidence rate.

¶ Indicates specific TNF-α antagonist users

§ Indicates those with last use of this TNF-α antagonist

† Restricted to patients using adalimumab alone or patients using etanercept alone, with or without non-biologic DMARDs.
